# Hepatic IRS1 and ß-catenin expression is associated with histological progression and overt diabetes emergence in NAFLD patients

**DOI:** 10.1007/s00535-018-1472-0

**Published:** 2018-05-10

**Authors:** Kenichiro Enooku, Mayuko Kondo, Naoto Fujiwara, Takayoshi Sasako, Junji Shibahara, Akira Kado, Kazuya Okushin, Hidetaka Fujinaga, Takeya Tsutsumi, Ryo Nakagomi, Tatsuya Minami, Masaya Sato, Hayato Nakagawa, Yuji Kondo, Yoshinari Asaoka, Ryosuke Tateishi, Kohjiro Ueki, Hitoshi Ikeda, Haruhiko Yoshida, Kyoji Moriya, Hiroshi Yotsuyanagi, Takashi Kadowaki, Masashi Fukayama, Kazuhiko Koike

**Affiliations:** 1 Department of Gastroenterology, Graduate School of Medicine, The University of Tokyo, 7-3-1 Hongo, Bunkyo-ku, Tokyo, 113-8655 Japan; 2 Department of Pathology, Graduate School of Medicine, The University of Tokyo, Tokyo, Japan; 3 Department of Clinical Laboratory Medicine, Graduate School of Medicine, The University of Tokyo, Tokyo, Japan; 4 Department of Infectious Diseases, Graduate School of Medicine, The University of Tokyo, Tokyo, Japan; 5 Department of Infection Control and Prevention, Graduate School of Medicine, The University of Tokyo, Tokyo, Japan; 6 Department of Diabetes and Metabolic Diseases, Graduate School of Medicine, The University of Tokyo, Tokyo, Japan; 7 Department of Pathology, Kyorin University, Mitaka, Japan; 8 Kawakita General Hospital, Tokyo, Japan

**Keywords:** NAFLD, IRS1, Hyperinsulinemia, Insulin resistance, Postprandial hyperglycemia

## Abstract

**Background:**

Nonalcoholic fatty liver disease (NAFLD) is a risk factor for type 2 diabetes. Our aim was to investigate the relationship between NAFLD and impaired glucose metabolism in terms of insulin receptor substrate 1 and 2 (IRS1 and IRS2) expression in the liver.

**Methods:**

Liver biopsy was performed at the University of Tokyo Hospital between November 2011 and March 2016 on 146 patients with NAFLD who were not being treated with any diabetes or dyslipidemia drugs. Among them, 63 underwent liver biopsy after an overnight fast, and 83 at 5 h after an oral glucose tolerance test (OGTT). Differences in messenger RNA (mRNA) levels of several glucose metabolism-related factors were determined and correlated with hepatic histological changes assessed by NAFLD activity score. We prospectively followed up with the patients until May 2017.

**Results:**

Hepatic necroinflammation was significantly correlated with serum insulin levels and inversely correlated with *IRS1* mRNA levels. In specimens obtained after an OGTT, hepatic necroinflammation and *IRS1* expression correlated significantly with both peripheral and hepatic insulin resistance. We also found that hepatic β-catenin and glucokinase mRNA levels were elevated in patients undergoing liver biopsy after an OGTT, especially in those with less hepatic necroinflammation and a lower degree of fibrosis. A prospective cohort study showed that ballooning is the most significant risk factor for developing diabetes.

**Conclusions:**

The decreased hepatic expression of *IRS1* and β-catenin in NAFLD is linked to histological progression such as ballooning, and might lead to diabetes as a result of impaired glucose metabolism.

**Electronic supplementary material:**

The online version of this article (10.1007/s00535-018-1472-0) contains supplementary material, which is available to authorized users.

## Introduction

Nonalcoholic fatty liver disease (NAFLD) is a leading cause of chronic liver disease and is strongly associated with metabolic syndrome. Type 2 diabetes mellitus (T2DM) is a systemic metabolic disorder closely related to NAFLD. Other studies have shown that NAFLD is frequently associated with insulin resistance [1–3], postprandial hyperglycemia [4], prediabetes, and undiagnosed T2DM [5, 6]. We have noticed that a substantial portion of patients with NAFLD subsequently develop diabetes within a few years.

However, the relationship between NAFLD and T2DM is complicated [7, 8]. Some animal models support the hypothesis that primary hepatic disease might underlie T2DM [9]. Some epidemiologic studies have shown a relationship between NAFLD and T2DM risk using surrogate NAFLD markers (transaminases and gamma-glutamyltransferase [GGT]) [1, 10, 11], semi-quantitative assessment of fatty liver (ultrasound, MRI) [5], and NAFLD algorithms [12]. Despite these indirect associations, a clear-cut link between NAFLD and T2DM has not been reported.

Mice specifically lacking the insulin receptor in the liver exhibit severe insulin resistance and hyperglycemia after feeding, indicating that hepatic insulin receptor signaling plays an essential role in the regulation of hepatic and systemic glucose homeostasis [13]. Insulin receptor substrates 1 and 2 (IRS1 and IRS2) predominantly mediate insulin signaling in the liver, and liver-specific IRS1- or IRS2-knockout mice exhibit insulin resistance in distinct ways [14]. We hypothesized that IRS1 and IRS2 expression would be abnormal in the livers of NAFLD patients, with the degree of abnormality related to the histologic severity of steatohepatitis.

In neoplasms, IRS1 expression is regulated by Wnt/β-catenin signaling [15]. In a skeletal muscle cell line, Wnt stimulation was shown to increase the transcription of *IRS1*, which is a direct transcriptional target of β-catenin [16]. β-catenin was also reported to regulate the establishment of hepatic metabolic zonation [17, 18]. These findings led us to study how hepatic β-catenin expression relates to steatohepatitis histology.

Based on IRS1 and IRS2 expression in the liver, we aimed to investigate the relationship between NAFLD and impaired glucose metabolism, focusing on hyperinsulinemia, insulin resistance, and postprandial hyperglycemia. In addition, we sought to confirm this relationship by investigating intrahepatic β-catenin and glucokinase (*GCK*) mRNA levels. Finally, we performed a prospective cohort study to investigate whether NAFLD histology is a significant risk factor for T2DM.

## Methods

### Patients

From November 2011 to March 2016, we prospectively recruited patients with clinically suspected NAFLD. When patients met each of the following criteria, we recommended they undergo liver biopsy: (1-A) liver transient elastography measured by Fibroscan^®^ above 7.0 kPa; (1-B) serum aspartate aminotransferase (AST) or alanine aminotransferase (ALT) levels above the normal upper limit for at least 6 months; (2) fatty liver diagnosed based on ultrasound examination showing an increase in hepatorenal contrast; (3) a history of alcohol consumption < 30 g/day for males and < 20 g/day for females; (4) seronegativity for hepatitis B virus surface antigen and hepatitis C virus antibody with the exclusion of primary biliary cirrhosis, autoimmune hepatitis, primary sclerosing cholangitis, drug-induced liver injury, Budd–Chiari syndrome, Wilson disease, hemosiderosis, and schistosomiasis. With regard to 1-A and 1-B, patients were included if they met at least one of the two criteria, as it was assumed that criterion 1-A reflected past liver injury and criterion 1-B reflected present liver injury. All patients provided informed written consent. Among patients with biopsy-proven NAFLD, only those who were not being treated with any diabetes drug including insulin and dyslipidemia drugs were included in the study.

### Data extraction

Comorbid illness and drug intake were recorded and body height and weight were measured on the day of admission. Blood samples for laboratory tests were taken on the morning of the liver biopsy, following an overnight fast. Blood tests included platelet counts, C-reactive protein, serum albumin, AST, ALT, GGT, total bilirubin, prothrombin time-international normalized ratio, ferritin, hyaluronic acid, fasting blood glucose (FBG), immunoreactive insulin (IRI), and hemoglobin A1c (HbA1c).

Until March 2014, we performed a 75-g oral glucose tolerance test (OGTT) after blood sampling on the morning of liver biopsy. Blood samples for this test were obtained at 0, 30, 60, 120, and 180 min for the measurement of glucose and IRI. Percutaneous liver biopsy was then performed 5 h after the OGTT. After April 2014, we performed liver biopsy after an overnight fast. Patients undergoing liver biopsy at 5 h after an OGTT were designated the glucose-loaded group and patients undergoing liver biopsy in the fasting state were referred to as the fasting group.

We used the homeostasis model assessment for insulin resistance (HOMA-IR) and the Matsuda Index of insulin sensitivity as surrogate indices in this study. HOMA-IR is dependent upon both peripheral and hepatic insulin sensitivity, the contribution of which differs between subjects with normal and elevated fasting glucose concentrations. In subjects with impaired fasting glucose or impaired glucose tolerance, hepatic insulin sensitivity is the most important determinant of HOMA-IR [19]. The Matsuda Index is a model that uses dynamic glucose and insulin values obtained during OGTT and provides a reasonable approximation of whole-body insulin sensitivity [20].

HOMA-IR and Matsuda indices were calculated as follows: HOMA-IR = FBG (mg/dL) × IRI (μU/mL)/405; Matsuda Index = 10,000/square root of (FBG [mg/dL] × IRI [μU/mL] × mean glucose [mg/dL] × mean IRI [μU/mL] during OGTT) [20]. Mean glucose and IRI were calculated from areas under the curves from the OGTT (using the trapezoidal method).

### Histologic assessment

Percutaneous liver biopsy was performed using a 16G needle with a biopsy specimen notch of 20 mm. All liver biopsy samples were examined before April 2016 by one experienced hepatopathologist (J.S.) who was blinded to the clinical data and study design. Liver histology was assessed according to Matteoni’s classification [21] and scored according to the Nonalcoholic Steatohepatitis Clinical Research Network criteria [22]. The NAFLD activity score (NAS) was the unweighted sum of steatosis, lobular inflammation, and hepatocellular ballooning scores. Nonalcoholic steatohepatitis (NASH) was defined as Matteoni type 3 or 4.

### RNA isolation, reverse transcription, and real-time reverse-transcription polymerase chain reaction

From liver biopsy specimens, a 3-mm-long sample was saved from each subject to measure gene expression [23]. Total RNA was isolated from the samples using a ReliaPrep™ RNA Cell Miniprep System (Promega, Madison, WI, USA). Reverse transcription was performed using a High Capacity cDNA Reverse Transcription Kit (Applied Biosystems, Foster City, CA, USA) to produce cDNA, according to the manufacturer’s instructions. Real-time PCR was performed using a StepOnePlus™ Sequence Detection System (Applied Biosystems) with the TaqMan Universal PCR Master Mix reagent (Applied Biosystems).

From liver tissue, we determined mRNA levels of *IRS1*, *IRS2*, β-catenin, *GCK*, and the housekeeping gene glyceraldehyde-3-phosphate dehydrogenase (*GAPDH*). Target values were normalized to the expression of *GAPDH*.

Primers and probes for *IRS1* (Hs.471508), *IRS2* (Hs.442344), β-catenin (Hs.476018), and *GCK* (Hs.1270) were purchased from an assay-on-demand facility (Applied Biosystems) as follows: *IRS1*, Hs00178563_m1; *IRS2*, Hs00275843_s1; β-catenin, Hs00355049_m1; *GCK*, Hs01564555_m1; *GAPDH*, 4326317E (Applied Biosystems).

### Analysis of clinical parameters, histology, and mRNA expression

We investigated the association between clinical parameters related to glucose metabolism and histology and intrahepatic *IRS1* and *IRS2* mRNA levels using univariate and multivariate analysis. Relationships among blood glucose levels at 120 min during OGTTs, the Matsuda Index, histology findings, intrahepatic *IRS1* and *IRS2* mRNA levels, and parameters related to liver injury and systemic inflammation were assessed. Because we found that glucose levels at 120 min significantly correlate with degree of lobular inflammation and ballooning and *IRS1* and *IRS2* mRNA expression, we stratified glucose concentrations during OGTTs using these four parameters. We then investigated the association between β-catenin and *GCK* mRNA expression and histology.

### Immunohistochemical analysis of β-catenin, IRS1, and GCK

To investigate β-catenin, IRS1, and GCK protein expression, immunohistochemistry was performed using paraffin-embedded histological sections of liver biopsy specimens. Antibodies included anti-β-catenin mouse monoclonal IgG (BD Biosciences, Franklin Lakes, NJ, USA), anti-IRS1 mouse monoclonal IgG (R&D systems, Minneapolis, MN, USA), and anti-GCK rabbit polyclonal IgG (Abcam, Burlingame, CA, USA) (Supplementary Table 3).

### Patient follow-up and analysis of T2DM risk factors

This study was cross-sectional, and from the results, we could not assess whether the degree of liver necroinflammation was directly correlated with T2DM risk. Therefore, we prospectively followed up on patients with HbA1c < 6.0% at the time of liver biopsy until May 2017 on an outpatient basis. Monthly follow-up was conducted to assess fasting blood glucose and HbA1c. T2DM was diagnosed in patients who met the ‘Criteria for the diagnosis of diabetes’ described in STANDARDS OF MEDICAL CARE IN DIABETES-2017 [24]. We performed univariate and multivariate analyses to investigate risk factors for developing T2DM.

### Statistics

Data processing and analysis were performed using S-PLUS version 8 (TIBCO Software, Inc., Palo Alto, CA, USA), with a two-tailed *P* value of < 0.05 considered statistically significant. For the analysis of patient characteristics, Fisher’s exact tests and Mann–Whitney *U* tests were used to investigate differences between fasting and glucose-loaded groups. Spearman’s rank correlation coefficient was used to examine correlations between two parameters selected from the liver histology score (lobular inflammation, hepatocyte ballooning, steatosis grade, fibrosis stage), physical variables (age, sex, body mass index), results of blood tests, and expression levels of *IRS1*, *IRS2*, β-catenin, and *GCK*. To investigate relationships between blood glucose levels at 120 min or the Matsuda Index of insulin sensitivity and each parameter, we used the Spearman’s rank correlation coefficient for univariate analysis and a linear regression model for multivariate analysis. For this, we explored model selection using the Akaike information criterion. For the analysis of T2DM risk factors, we used univariate and multivariate Cox hazard regression analysis.

### Ethical guidelines

This study was conducted according to ethical guidelines relevant to epidemiologic research promulgated by the Japanese Ministry of Education, Culture, Sports, Science and Technology and the Ministry of Health, Labour, and Welfare. The study design was described in a comprehensive protocol prepared by the Department of Gastroenterology, the University of Tokyo Hospital, and was approved by the University of Tokyo Medical Research Center Ethics Committee (approval number 3955).

## Results

### Patient characteristics

The total number of patients who met the above inclusion criteria was 146 (thus, 146 patients were included in this study.) Basic characteristics including liver histology are detailed in Tables 1 and 2. Liver biopsy was performed for 83 patients 5 h post-OGTT (glucose-loaded group) before March 2014, and in 63 individuals following overnight fasting (fasting group) thereafter. For three patients in the fasting group and four in the glucose-loaded group, we could not measure liver stiffness due to obesity. There were no significant differences in baseline characteristics between fasting and glucose-loaded groups.

**Table 1:** Patient characteristics stratified by timing of liver biopsy in patients with nonalcoholic fatty liver disease

Parameter	Patients undergoing liver biopsy after an overnight fast (*N* = 63)	Patients undergoing liver biopsy at 5 h after an OGTT (*N* = 83)	*P*
Male/female^†^	40/23	50/33	0.73
Age (years)^‡^	45.1 (37.7–55.5)	51.2 (41.1–65.4)	0.063
BMI (kg/m^2^)^‡^	28.6 (24.8–31.6)	27.7 (25.6–30.2)	0.88
Liver stiffness (kPa)^‡^	7.6 (5.9–10.5)	7.7 (6.1–11.8)	0.45
Hypertension on treatment (%)^†^	13 (20.6)	24 (28.9)	0.19
Platelet count (× 10^4^/μL)^‡^	24.1 (19.8–27.5)	22.2 (17.5–25.5)	0.23
CRP (mg/dL)	0.13 (0.06–0.33)	0.10 (0.06–0.24)	0.33
Albumin (g/dL)^‡^	4.0 (3.7–4.2)	4.1 (3.8–4.3)	0.21
AST (U/L)^‡^	38 (27–49.5)	41 (31–61)	0.23
ALT (U/L)^‡^	65 (34–94.5)	61 (40–94.5)	0.80
AST to ALT ratio^‡^	0.62 (0.48–0.79)	0.72 (0.51–0.91)	0.11
GGT (U/L)^‡^	69 (46.5–131)	63 (39–89.5)	0.14
Total bilirubin (mg/dL)^‡^	0.9 (0.7–1.2)	0.9 (0.7–1.2)	0.63
HDL cholesterol (mg/dL)^‡^	44.9 (37.0–54.1)	48.4 (39.8–55.7)	0.21
LDL cholesterol (mg/dL)^‡^	129 (106–153)	124 (105–132)	0.16
Triglyceride (mg/dL)^‡^	130 (96–205)	117 (98.5–155.5)	0.23
PT-INR^‡^	0.94 (0.90–1.01)	0.94 (0.90–0.99)	0.72
Hyaluronic acid (ng/mL)^‡^	18.7 (10.0–36.3)	22.3 (13.6–45.7)	0.087
Fasting blood glucose (mg/dL)^‡^	91 (86–100)	90 (85–99)	0.68
HbA1c (NGSP) (%)^‡^	5.8 (5.5–6.2)	5.6 (5.4–6.1)	0.24

Values are presented as *N*, *N* (%), or median (P25, P75)

*AST* aspartate aminotransferase, *ALT* alanine aminotransferase, *BMI* body mass index, *CRP* C-reactive protein, *GGT* gamma-glutamyltransferase, *HbA1c* hemoglobin A1c, *HDL* high density lipoprotein, *LDL* low density lipoprotein, *OGTT* oral glucose tolerance test, *PT-INR* prothrombin time-international normalized ratio

^†^Fisher’s exact tests were used to investigate the difference in each parameter between patients undergoing liver biopsy after an overnight fast or at 5 h post-OGTT

^‡^Mann–Whitney *U* tests were used to investigate the difference for each parameter between patients undergoing liver biopsy after an overnight fast or at 5 h post-OGTT

**Table 2:** Steatohepatitis histology stratified by timing of liver biopsy in patients with nonalcoholic fatty liver disease

Parameter		Patients undergoing liver biopsy after an overnight fast (*N* = 63)	Patients undergoing liver biopsy at 5 h after an OGTT (*N* = 83)	*P*
Matteoni classification				
Type I: steatosis alone		3 (4.8)	7 (8.4)	0.20
Type II: steatosis with inflammation		19 (30.2)	15 (18.1)	
Type III–IV: steatosis with ballooning and/or fibrosis		41 (65.1)	61 (73.5)	
NAS score				
0–2 (%)		3 (4.8)	8 (9.6)	0.11
3–4 (%)		35 (55.6)	32 (38.6)	
5–8 (%)		25 (39.7)	43 (51.8)	
Lobular inflammation				
None (%)	0	4 (6.3)	8 (9.6)	0.87
< 2 (%)	1	45 (71.4)	55 (66.3)	
2–4 (%)	2	13 (20.6)	19 (22.9)	
> 4 (%)	3	1 (1.6)	1 (1.2)	
Ballooning				
None (%)	0	22 (34.9)	22 (26.5)	0.24
Few (%)	1	36 (57.1)	47 (56.6)	
Many (%)	2	5 (7.9)	14 (16.9)	
Steatosis grade				
< 5% (%)	0	0 (0.0)	0 (0.0)	0.39
5–33% (%)	1	27 (42.9)	43 (51.8)	
34–66% (%)	2	21 (33.3)	27 (32.5)	
> 67 (%)	3	15 (23.8)	13 (15.7)	
Fibrosis stage				
0 (%)		14 (22.2)	15 (18.1)	0.12
1 (%)		31 (49.2)	28 (33.7)	
2 (%)		10 (15.9)	19 (22.9)	
3 (%)		4 (6.3)	15 (18.1)	
4 (%)		4 (6.3)	6 (7.2)	

Values are presented as *N*, *N* (%). Fisher’s exact tests were used to investigate the difference for each parameter between patients undergoing liver biopsy after an overnight fast or at 5 h post-OGTT

*OGTT* oral glucose tolerance test, *NAS* the NAFLD activity score

### Relationship between histology and glucose metabolism variables

**Fig. 1:**
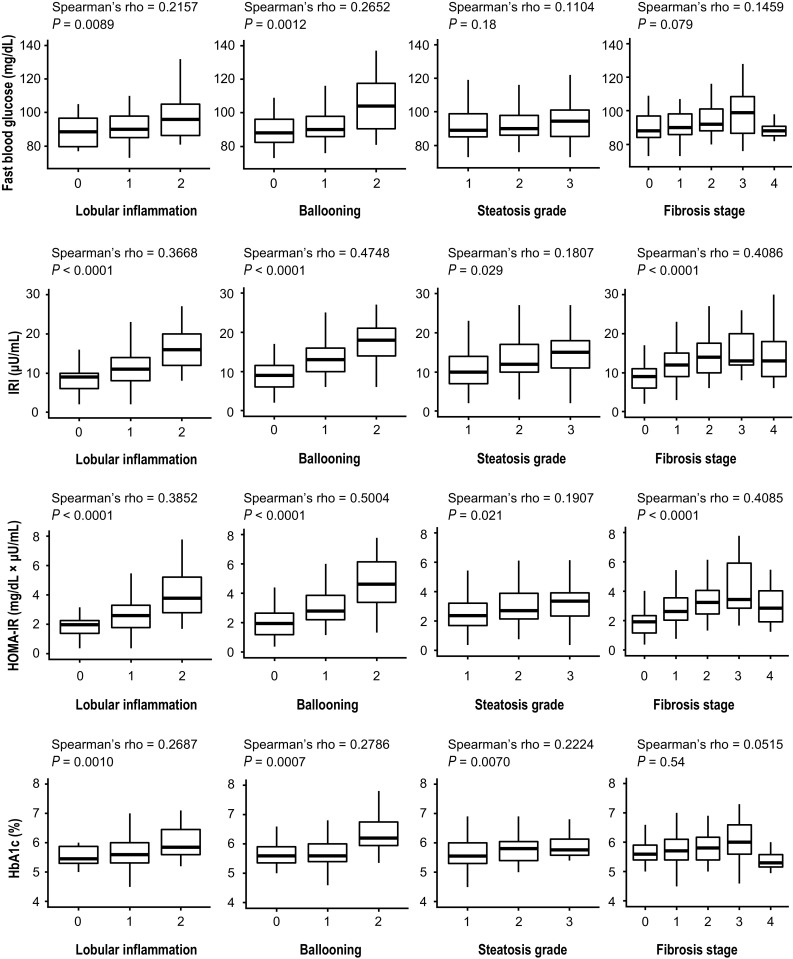
Relationship between steatohepatitis histology and glucose metabolism parameters, all of which were very strongly correlated with lobular inflammation and ballooning. Spearman’s rank correlation coefficient was used to examine these correlations. *IRI* immunoreactive insulin, *HOMA-IR* homeostasis model assessment for insulin resistance, *HBA1c* hemoglobin A1c

Figure 1 shows the relationship between liver histology and glucose metabolism variables. FBG was significantly correlated with lobular inflammation and ballooning, albeit less so than IRI, which was strongly correlated with lobular inflammation, ballooning, and fibrosis. HOMA-IR correlated significantly with lobular inflammation, ballooning, and fibrosis. HbA1c was significantly correlated with lobular inflammation, ballooning, and steatosis grade.

### Relationship between *IRS1/IRS2* mRNA expression and steatohepatitis histology and glucose metabolism variables

**Fig. 2:**
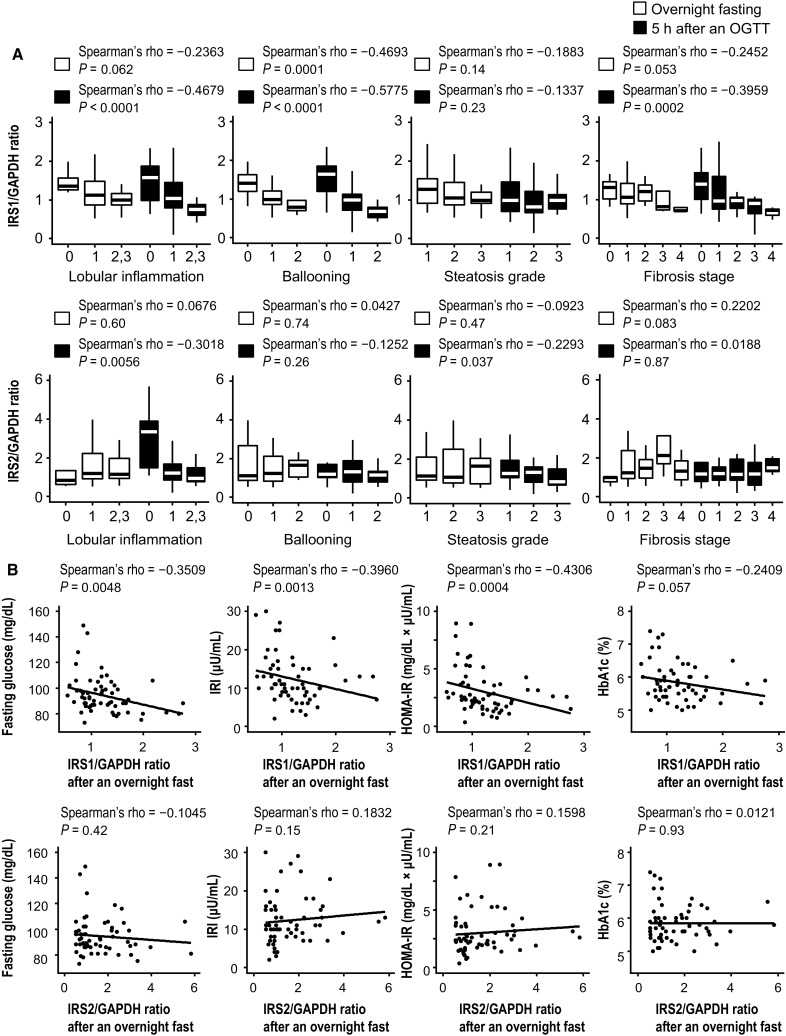
Relationship between *IRS1/2* levels and steatohepatitis histology and metabolism parameters. Spearman’s rank correlation coefficient was used to examine these correlations. **a** *IRS1* and *IRS2* mRNA expression and steatohepatitis histology. **b** *IRS1* and *IRS2* mRNA expression and glucose metabolism-related parameters. *IRI*, immunoreactive insulin, *HOMA-IR* homeostasis model assessment for insulin resistance, *HBA1c* hemoglobin A1c

*IRS1* mRNA levels were lower with increasing degrees of hepatic necroinflammatory activity (Fig. 2a). In particular, ballooning had a strong negative correlation with *IRS1* levels; this was stronger in the glucose-loaded group. *IRS2* mRNA levels were significantly correlated with lobular inflammation and steatosis grade in the glucose-loaded group, but not in the fasting group.

Figure 2b shows the comparison between *IRS1* and *IRS2* mRNA levels and glucose metabolism variables in the fasting group. *IRS1* levels were significantly correlated with FBG, IRI, and HOMA-IR, whereas *IRS2* levels showed no significant correlation with any parameters.

### Relationship between blood glucose or Matsuda Index and physical/clinical parameters or steatohepatitis histology

Table 3 shows the relationship between blood glucose levels at 120 min during OGTTs or the Matsuda Index and physical and clinical parameters or steatohepatitis histology in the glucose-loaded group. Blood glucose levels at 120 min during the OGTT were significantly correlated with severity of steatohepatitis and *IRS1* and *IRS2* mRNA levels. Based on multivariate analysis, lobular inflammation was most significantly correlated with blood glucose levels at 120 min.

**Table 3:** Relationship between blood glucose at 120 min after an OGTT or the Matsuda Index and each parameter in patients with nonalcoholic fatty liver disease undergoing liver biopsy at 5 h after OGTTs (*N* = 83)

Parameter	Blood glucose at 120 min after OGTT				The Matsuda Index			
	Spearman’s rank correlation		Linear regression model		Spearman’s rank correlation		Linear regression model	
	Spearman’s rho	*P*	Regression coefficient (SE)	*P*	Spearman’s rho	*P*	Regression coefficient (SE)	*P*
Male/female	0.0356	0.75			− 0.1371	0.22		
Age (years)	0.0241	0.83			− 0.2781	0.012		
BMI (kg/m^2^)	0.1099	0.33			− 0.1769	0.11		
Liver stiffness (kPa)	0.1036	0.38			− 0.2682	0.020		
Platelet count (× 10^4^/μL)	− 0.0261	0.82			0.0447	0.69		
CRP (mg/dL)	0.3210	0.003	55.49 (31.51)	0.082	− 0.4351	< 0.001	− 2.418 (1.232)	0.053
Albumin (g/dL)	0.0332	0.77			0.2170	0.051		
AST (U/L)	0.2848	0.010			− 0.2233	0.045		
ALT (U/L)	0.3754	< 0.001			− 0.1472	0.19		
AST to ALT ratio	− 0.1451	0.20			− 0.0925	0.41		
GGT (U/L)	0.4249	< 0.001	0.264 (0.093)	0.006	− 0.0913	0.42		
Total bilirubin (mg/dL)	0.0479	0.67			− 0.0323	0.77		
PT-INR (%)	− 0.0864	0.44			− 0.1629	0.15		
Hyaluronic acid (ng/mL)	0.0922	0.41			− 0.2792	0.012		
Lobular inflammation	0.3764	< 0.001	37.25 (12.51)	0.004	− 0.1481	0.18		
Ballooning	0.3009	0.006	15.52 (11.26)	0.17	− 0.2799	0.011		
Steatosis grade	0.2448	0.028			− 0.0907	0.42		
Fibrosis stage	0.1182	0.29			− 0.3622	< 0.001	− 0.439 (0.213)	0.043
IRS-1/GAPDH ratio at 5 h after OGTT	− 0.2701	0.015			0.2911	0.006	0.947 (0.341)	0.007
IRS-2/GAPDH ratio at 5 h after OGTT	− 0.2229	0.046			0.1343	0.23		

Parameters included were physical variables, markers chiefly related to liver injury and systemic inflammation, and steatohepatitis histology. We used Spearman’s rank correlation coefficients for univariate analysis and a linear regression model for multivariate analysis. For the multivariate analysis, we explored model selection using the Akaike information criterion

*AST* aspartate aminotransferase, *ALT* alanine aminotransferase, *BMI* body mass index, *CRP* C-reactive protein, *GGT* gamma-glutamyltransferase, *HbA1c* hemoglobin A1c, *HDL* high density lipoprotein, *LDL* low density lipoprotein, *OGTT* oral glucose tolerance test, *PT-INR* prothrombin time-international normalized ratio

To assess whole-body insulin sensitivity, we calculated the Matsuda Index. Hepatocellular ballooning and *IRS1* mRNA levels were significantly correlated with this parameter. Of note, based on multivariate analysis, *IRS1* levels were most significantly correlated with Matsuda Index.

**Fig. 3:**
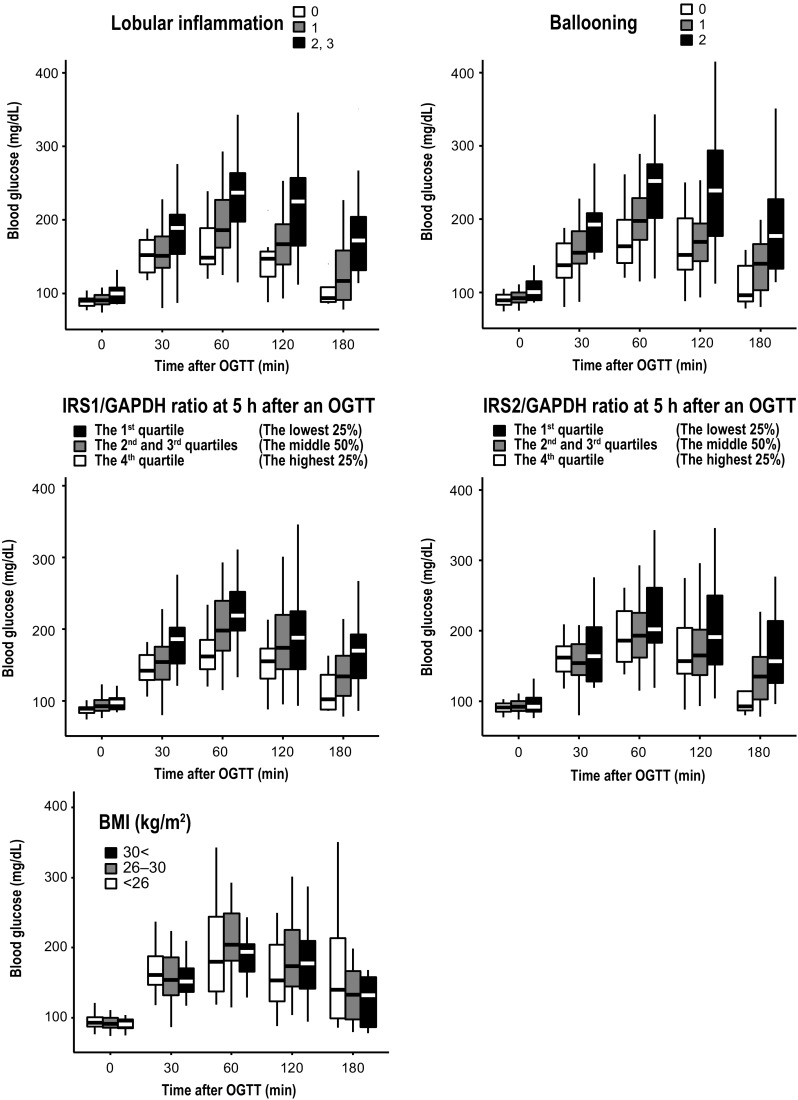
Glucose concentrations during 75-g oral glucose tolerance tests (OGTTs) stratified based on degree of lobular inflammation and ballooning and *IRS1* and *IRS2* mRNA levels

We then stratified glucose concentrations during the OGTTs based on degree of lobular inflammation and ballooning and *IRS1* and *IRS2* mRNA levels (Fig. 3). Peak glucose concentrations during the OGTTs were higher and delayed in patients with more severe necroinflammation and lower *IRS1* and *IRS2* mRNA levels. Glucose concentrations decreased to the normal range faster in patients with less severe steatohepatitis and higher *IRS1* and *IRS2* mRNA levels. Although *IRS1* mRNA levels were significantly correlated with blood glucose throughout the OGTT, *IRS2* mRNA levels were significantly correlated with blood glucose only during the last phase (120–180 min) of the OGTT (Supplementary Table 1).

### Relationship between β-catenin and *GCK* and steatohepatitis histology

**Fig. 4:**
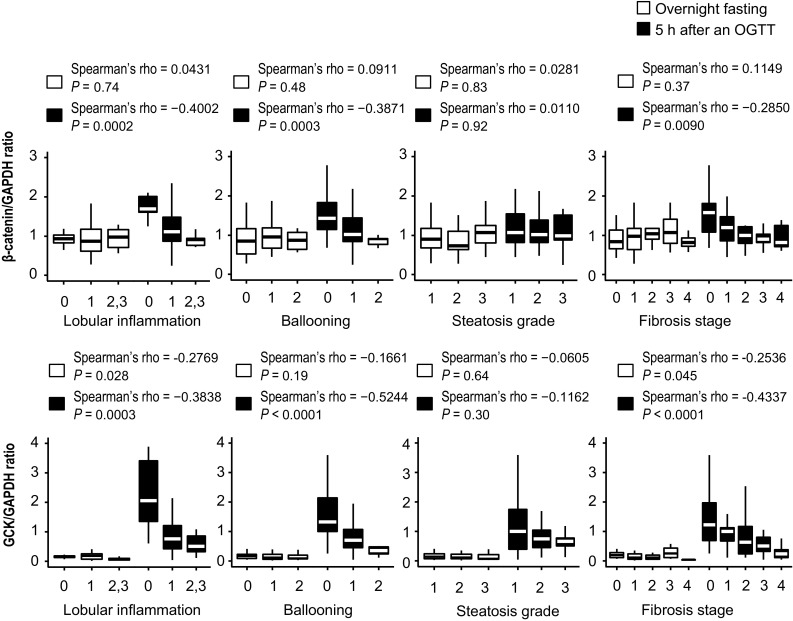
Relationship between β-catenin or *GCK* mRNA levels and steatohepatitis histology. Spearman’s rank correlation coefficient was used to examine these correlations. Hepatic β-catenin and *GCK* mRNA levels were measured in specimens from patients undergoing liver biopsy at 5 h after oral glucose tolerance tests (OGTTs)

Figure 4 shows β-catenin and *GCK* mRNA levels in relation to steatohepatitis histology. β-Catenin mRNA levels were elevated in the glucose-loaded group, but not elevated in the fasting group. This elevation was greater in patients with less severe steatohepatitis and a lower fibrosis stage.

*GCK* mRNA levels were very low (almost zero) in the fasting state, but were strikingly elevated in the glucose-loaded group. Remarkably, this elevation was strongly and significantly correlated with less severe steatohepatitis.

### Immunohistochemical analysis of β-catenin and IRS1 in liver biopsy specimens

**Fig. 5:**
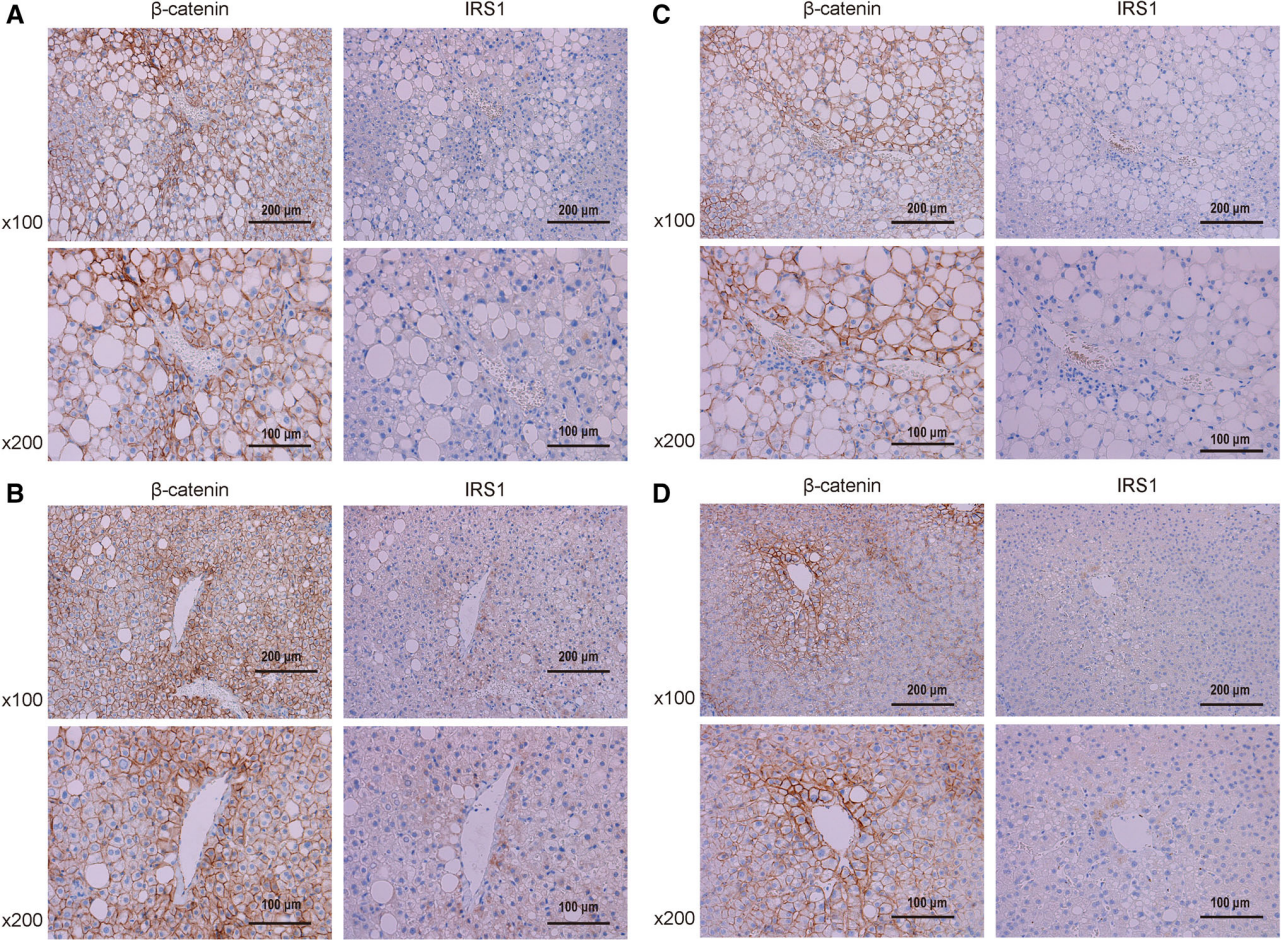
Immunohistochemical staining of liver biopsies from representative nonalcoholic fatty liver disease patients. Patients A and B underwent liver biopsy 5 h after oral glucose tolerance tests (OGTT), whereas patients C and D underwent liver biopsy in a fasting state. **a** Patient A was a 44-year-old male with severe ballooning. **b** Patient B was a 31-year-old male, with no ballooning. **c** Patient C was a 47-year-old male and had severe ballooning. **d** Patient D was a 45-year-old male and had no ballooning. Liver biopsies were stained for β-catenin and IRS1. Positive immunoreactivity appears brown. Original magnification, ×100 or ×200

Immunohistochemical staining of liver biopsies from representative NAFLD patients who underwent liver biopsy 5 h after the OGTT is shown for patient A and B (Fig. 5a, b). Patient A was a 44-year-old male with severe ballooning (lobular inflammation, 2; ballooning, 2; steatosis grade, 2; fibrosis stage, 1a). Patient B was a 31-year-old male with no ballooning (lobular inflammation, 1; ballooning, 0; steatosis grade, 2; fibrosis stage, 1c). Liver biopsies were stained for β-catenin and IRS1. Activated β-catenin is known to be distributed in the cytoplasm and nucleus. In patient B, cells surrounding the central vein with cytoplasmic β-catenin distribution appeared to have high IRS1 expression. In patient A samples, total β-catenin expression was lower than that in patient B, and the number of IRS1-expressing hepatocytes was small.

Immunohistochemical staining of liver biopsies from representative NAFLD patients who underwent liver biopsy in the fasting state is shown for patients C and D (Fig. 5c, d). Patient C was a 47-year-old male with severe ballooning (lobular inflammation, 2; ballooning, 2; steatosis grade, 2; fibrosis stage, 1b). Patient D was a 45-year-old male with no ballooning (lobular inflammation, 1; ballooning, 0; steatosis grade, 1; fibrosis stage, 0). β-Catenin expression was lower in patients C and D than in glucose-loaded patients. In patient D, β-catenin and IRS1 expression was lower than that in patient B. In patient C, β-catenin expression was lower than that in patient A, and IRS1 expression was seldom observed.

Supplementary Figure 1 shows liver biopsies stained for GCK. GCK was highly expressed in pericentral hepatocytes, similar to IRS1. GCK expression was higher in the glucose-loaded group, especially in patient B.

### Analysis of T2DM risk factors

HbA1c was less than 6.0% at the time of liver biopsy in 107 patients. The median follow-up time was 2.33 years (IQR 1.33–3.66 years). Supplementary Table 2 shows the risk factors for developing T2DM, evaluated by univariate and multivariate Cox proportional hazard regression. Based on univariate analysis, BMI (body mass index), AST, ALT, ballooning, and steatosis grade were significant risk factors. Among these, based on multivariate analysis, ballooning was the only significant risk factor. Supplementary Figure 2 shows the cumulative incidence of diabetes stratified by ballooning in patients with HbA1c < 6.0% at liver biopsy. Thus, patients with hepatocellular ballooning were clearly at a higher risk of developing diabetes.

## Discussion

In the present study, FBG and serum insulin levels were strongly correlated with lobular inflammation and ballooning, rather than with steatosis grade. Consequently, HOMA-IR was more strongly associated with hepatic necroinflammatory activity than with steatosis grade. Because hepatic insulin sensitivity is the most important determinant of HOMA-IR in patients with impaired fasting glucose or impaired glucose tolerance, this result suggested that hepatic insulin sensitivity is dependent on the severity of hepatic necroinflammatory activity. We found that in patients with NAFLD, hepatic *IRS1* mRNA levels were significantly lower with higher degrees of hepatic necroinflammatory activity. In addition, FBG, IRI, and HOMA-IR were inversely correlated with hepatic *IRS1* mRNA levels. This suggests that in NAFLD, hyperinsulinemia, and hepatic insulin resistance result from decreased hepatic IRS1 expression associated with hepatic necroinflammatory activity. Decreased IRS1 in the liver can cause the fasting hyperinsulinemia often observed in patients with NAFLD, since higher serum insulin levels are needed to suppress gluconeogenesis in the liver. Increased hepatic gluconeogenesis occurs in NAFLD [25, 26], and hepatic insulin resistance is associated with increased insulin secretion required to regulate the normal rate of hepatic glucose production [27–29]. Thus, the continuous burden imposed on pancreatic beta cells is thought to be a predisposing factor for T2DM development [30].

Concerning the association between liver diseases and impaired glucose metabolism, we previously reported that hepatic insulin resistance is observed in transgenic HCV-harboring mice that develop hepatic steatosis and finally hepatocellular carcinoma [31–33], which is a commonly observed pathogenesis in HCV-infected human livers. In these mice, IRS1 expression is unchanged, but tyrosine phosphorylation of IRS1 upon insulin stimulation is impaired due to increased TNF-α expression. A high-fat diet was shown to lead to the development of overt diabetes. Considering this, although the exact mechanism of IRS1 dysfunction is different in distinct conditions, IRS1 might be important for hepatic insulin resistance and finally T2DM progression.

We also demonstrated that blood glucose levels at 120 min during the OGTT were significantly correlated with degree of lobular inflammation and ballooning and the *IRS1* and *IRS2* expression in the liver, and that glucose concentration patterns during the OGTTs were clearly separated by degree of lobular inflammation and ballooning and *IRS1* and *IRS2* mRNA levels. During the late phase (60–180 min) of the OGTT, hepatic glucose production is suppressed and peripheral glucose uptake is activated [34]. Thus, OGTT curves at 60–180 min are thought to reflect peripheral insulin resistance. Additionally, the Matsuda Index, a surrogate marker reflecting whole-body insulin sensitivity, was strongly correlated with hepatic *IRS1* mRNA levels. These results suggest that in patients with NAFLD, hepatic insulin resistance is accompanied by peripheral insulin resistance. This study excluded patients diagnosed with DM; therefore probably insulin secretion is maintained in these patients. Actually, patients with high BMI tend to present hyperinsulinemia, which might suppress increase of blood glucose. We speculate that this is the reason why BMI did not correlate with glucose concentration patterns during the OGTTs. Hepatic IRS1 and IRS2 expression thus had a stronger and more direct influence than BMI on glucose concentration patterns during the OGTTs.

Supplementary Table 1 shows the relationship between blood glucose levels during OGTT and *IRS2* mRNA levels at 5 h post-OGTT. Hepatic *IRS2* mRNA levels after an overnight fast were not correlated with FBG, IRI, or HOMA-IR. However, the relationship between blood glucose levels and *IRS2* mRNA levels at 5 h post-OGTT became gradually closer as time elapsed after glucose loading. At 5 h post-OGTT blood glucose levels at 180 min were more strongly correlated with *IRS2* mRNA levels than with *IRS1*. These results suggested that in the fasting state, IRS1 is more important for hepatic insulin signaling than IRS2, and that after glucose loading IRS2 is as important as IRS1. Kubota et al. described how IRS1 and IRS2 levels change in response to fasting and refeeding in normal mice [14]. They reported that IRS1 mRNA and protein levels were virtually unchanged and unaffected by food intake, whereas IRS2 mRNA and protein levels increased during fasting and rapidly decreased after refeeding. Sufficient expression of hepatic IRS2 during fasting might be necessary to lower blood glucose rapidly after feeding. In the present study, *IRS2* expression 5 h post-OGTT was significantly lower in patients with higher lobular inflammation and steatosis grade. This suggests that patients with severe steatohepatitis should maintain sufficient time between meals to restore hepatic IRS2 levels, because the restoration of IRS2 after meals might be delayed in NASH patients.

We also found that hepatic β-catenin expression was elevated in the glucose-loaded group, and that this elevation was even greater in patients with less severe steatohepatitis and lower fibrosis stage. β-catenin is one of the key molecules regulating metabolic zonation in the liver [35, 36]. There is a gradient of β-catenin expression along the portocentral axis in liver lobules. In periportal hepatocytes, the absence of β-catenin mediates the expression of periportal genes such as those involved in lipogenesis and gluconeogenesis. In addition, β-catenin activates Wnt-responsive elements on the regulatory regions of β-catenin-induced genes such as those involved in glucose uptake, glycogen synthesis, and glycolysis. Insulin receptor protein and IRS1 are predominantly localized to the pericentral zone based on the need for glucose uptake for glycogen synthesis and glycolysis [37]. Thus, IRS1 is highly expressed in pericentral hepatocytes under the control of β-catenin. Elevated GCK levels were thought to reflect enhanced insulin signaling mediated by IRS1 upregulation. We speculate that the significant correlation between *IRS1* mRNA levels and liver histology is caused by the response of β-catenin mRNA expression to glucose load, explaining the stronger association between *IRS1* mRNA levels and liver histology in the glucose-loaded group. Concerning β-catenin expression, a difference was observed between groups. Specifically, mRNA levels of β-catenin in the histologically advanced, glucose-loaded group were similar to those in both the advanced and non-advanced fasting groups, whereas protein expression was more prominent compared to that in both of these groups. Cellular β-catenin is also controlled by its ubiquitination and proteasomal degradation. Thus, differences in β-catenin mRNA levels might not precisely reflect protein levels. However, immunohistochemical staining showed that membranous expression of β-catenin in the glucose-loaded group was higher than that in the fasting group. In addition, this upregulation was more evident in patients with less severe steatohepatitis and a lower fibrosis stage compared to that in individuals with histologically advanced stage, similar to that observed for mRNA levels. We emphasize that upregulation of β-catenin, and subsequently IRS1, by glucose loading was suppressed by histological progression, which leads to impaired glucose metabolism.

These results indicate that glucose uptake is downregulated in the hepatocytes of NASH-affected livers; to our knowledge, this was previously unreported. Bock et al. reported that postprandial hyperglycemia in individuals with early diabetes is due to lower rates of glucose disappearance rather than increased meal appearance or impaired suppression of endogenous glucose production, regardless of fasting glucose levels [38]. Thus, postprandial hyperglycemia in patients with early diabetes can be at least partly explained by the results of NASH-affected livers.

Patatin-like phospholipase domain-containing protein 3 (*PNPLA3*) polymorphisms have been confirmed to be associated with NAFLD histological changes.[39, 40] While there are few studies reporting a direct association between PNPLA3 and the Wnt/β-catenin pathway, there are reports that *PNPLA3* gene expression is activated by sterol-regulatory element-binding protein (SREBP) 1c in human hepatocytes [41, 42]. Moreover, some studies indicate that expression of SREBP1c is suppressed by the activation of the Wnt/β-catenin pathway [43, 44]. It is well known that the increased expression of PNPLA3 is associated with hepatic steatosis. Based on the above, the decreased hepatic expression of β-catenin in NAFLD might be linked to increased expression of SREBP1c and PNPLA3.

The dysregulation of β-catenin levels and localization and constitutive activation of β-catenin-regulated gene expression occur in many cancers, including hepatocellular carcinoma (HCC). Expression of *IRS1* can be directly activated by β-catenin, and *IRS1* is highly expressed in many cancers with constitutive stabilization of β-catenin. Sakurai et al. reported that the upregulation of *IRS1* by Wnt/β-catenin signaling plays a crucial role in the progression of HCC [45]. At the same time, Zhang et al. reported that the loss of β-catenin impairs the liver’s ability to counteract *N*-nitrosodiethylamine (DEN)-induced oxidative stress and enhances tumorigenesis [46]. Rignall et al. found that hepatocyte-specific knockout of β-catenin enhances phenobarbital-induced hepatocarcinogenesis and induces a pre-cirrhotic phenotype in the mouse liver [47]. Furthermore, in chronic alcoholic liver disease, β-catenin signaling is downregulated, which enhances oxidative stress and is associated with severe liver damage [48, 49]. Based on these findings, the downregulation of β-catenin in the liver may exacerbate hepatic damage induced by various carcinogens, such as DEN, phenobarbital, and ethanol, and promote hepatic carcinogenesis. We speculate that the inactivation of β-catenin signaling in NASH hepatocytes may enhance tumor initiation in the liver. However, it is notable that almost all previously reported liver biopsy samples were collected during a fasting state, in which β-catenin expression is very low in background liver. Therefore, further investigation of β-catenin expression in background liver and HCC in a glucose-loaded state is required.

Cox proportional hazard regression revealed that patients with ballooning had a significantly higher risk of developing T2DM. We found that hepatic *IRS1* expression is much lower in patients with this condition. In NASH patients, decreased *IRS1* expression in the liver could lead to T2DM due to impaired glucose metabolism including fasting hyperinsulinemia and postprandial hyperglycemia. Whether NASH and T2DM are causally related or merely associated with metabolic syndrome remains to be established; here, we provided convincing evidence that NASH is one of the most important risk factors for developing T2DM.

Sajan et al. reported that hepatic *IRS1* levels diminished as BMI increased [50]. They concluded that hepatic insulin resistance progressed with BMI due to decreases in hepatic *IRS1* expression. However, in the present study, HOMA-IR, reflecting hepatic insulin sensitivity, was more strongly correlated with hepatic necroinflammatory activity than with steatosis grade. The degree of lobular inflammation and ballooning and *IRS1* mRNA levels, but not BMI, clearly separated glucose concentration patterns during the OGTTs. In addition, a prospective cohort study showed that hepatocyte ballooning is the most significant risk factor for developing diabetes. We suggest that BMI and hepatic triglyceride levels are less important for hepatic insulin resistance and the development of T2DM than hepatic necroinflammatory activity in NAFLD. We showed here elevated β-catenin expression after glucose loading, which was not correlated with steatosis grade. We consider that β-catenin upregulation plays an important role in dynamic alterations in the liver after feeding, including changes in *IRS1* expression; this is the key finding of the present study.

This study has several limitations. The number of patients was not large. Although we set the interval between the OGTT and liver biopsy at 5 h, this might not be the best time to observe differences in expression. Further studies are needed to identify the best intervals for these observations. The mechanisms underlying β-catenin upregulation after a glucose load and the relationship between β-catenin mRNA levels and liver histology remain undetermined.

In conclusion, hepatic IRS1 expression was inversely correlated with NAFLD histologic changes and could be a cause of glucose metabolic disorders, which are typically observed with NAFLD. Elevated β-catenin expression after glucose loading might explain changes in *IRS1* levels. Furthermore, our prospective cohort study suggested that ballooning was one of the most important risk factors for the development of overt diabetes. This study suggests that decreased hepatic expression of *IRS1* and β-catenin in NASH is linked to histological progression, which might lead to T2DM due to insulin resistance.

## Electronic supplementary material

Below is the link to the electronic supplementary material.

Supplementary Figure 1. Immunohistochemical staining of liver biopsies from representative nonalcoholic fatty liver disease patients. Patient A and B underwent liver biopsy 5 h after oral glucose tolerance tests (OGTTs), whereas Patient C and D underwent liver biopsy in a fasting state. (A) Patient A was a 44-year-old male with severe ballooning. (B) Patient B was a 31-year-old male with no ballooning. (C) Patient C was a 47-year-old male and had severe ballooning. (D) Patient D was a 45-year-old male and had no ballooning. Liver biopsies were stained for GCK. Positive immunoreactivity appears brown. Original magnification, × 100 or × 200. (JPEG 2504 kb)

Supplementary Figure 2. Cumulative incidence of diabetes in patients with hemoglobin A1c (HbA1c) < 6.0% at liver biopsy stratified by ballooning. Median of follow-up period was 2.33 years (IQR 1.33–3.66). The median and interquartile range of fasting blood glucose and HbA1c were 88.0 (81.5–92.5) mg/dl and 5.5 (5.3–5.8) % for patients with ballooning 0, 88.0 (85.0–94.0) mg/dl and 5.5 (5.3–5.7) % for patients with ballooning 1, and 92.0 (85.0–97.0) mg/dl and 5.9 (5.6–5.9) % for patients with ballooning 2. A Kruskal–Wallis test showed no significant difference in fasting blood glucose and HbA1c among the three groups. (PDF 110 kb)

Supplementary material 3 (DOCX 18 kb)
