# Supplementary figures and images for: Hepatic IRS1 and ß-catenin expression is associated with histological progression and overt diabetes emergence in NAFLD patients
Source: J Gastroenterol. 2018 May 10;53(12):1261–75. doi: 10.1007/s00535-018-1472-0 (PMC6244858; doi:10.1007/s00535-018-1472-0)

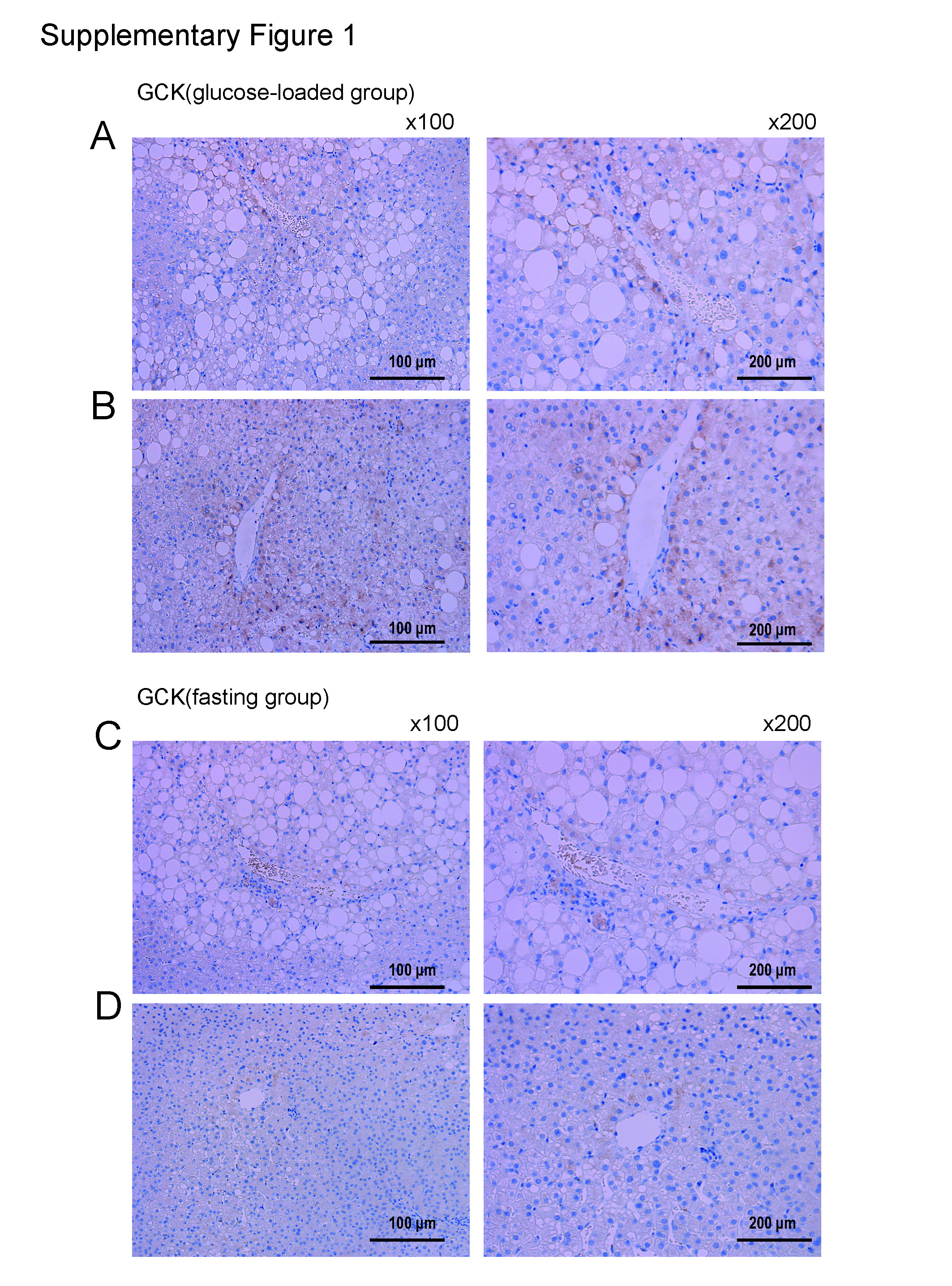

Supplement: Supplementary file 1 — Supplementary Figure 1. Immunohistochemical staining of liver biopsies from representative nonalcoholic fatty liver disease patients. Patient A and B underwent liver biopsy 5 h after oral glucose tolerance tests (OGTTs), whereas Patient C and D underwent liver biopsy in a fasting state. (A) Patient A was a 44-year-old male with severe ballooning. (B) Patient B was a 31-year-old male with no ballooning. (C) Patient C was a 47-year-old male and had severe ballooning. (D) Patient D was a 45-year-old male and had no ballooning. Liver biopsies were stained for GCK. Positive immunoreactivity appears brown. Original magnification, × 100 or × 200. (JPEG 2504 kb) [file 535_2018_1472_MOESM1_ESM.jpg]

Supplementary Figure 2

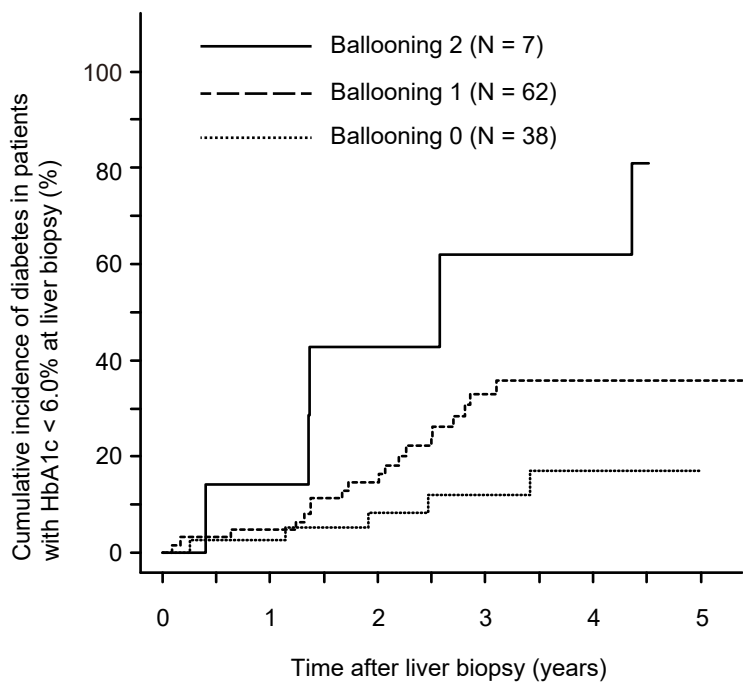

Supplement: Supplementary file 2 — Supplementary Figure 2. Cumulative incidence of diabetes in patients with hemoglobin A1c (HbA1c) < 6.0% at liver biopsy stratified by ballooning. Median of follow-up period was 2.33 years (IQR 1.33–3.66). The median and interquartile range of fasting blood glucose and HbA1c were 88.0 (81.5–92.5) mg/dl and 5.5 (5.3–5.8) % for patients with ballooning 0, 88.0 (85.0–94.0) mg/dl and 5.5 (5.3–5.7) % for patients with ballooning 1, and 92.0 (85.0–97.0) mg/dl and 5.9 (5.6–5.9) % for patients with ballooning 2. A Kruskal–Wallis test showed no significant difference in fasting blood glucose and HbA1c among the three groups. (PDF 110 kb) [file 535_2018_1472_MOESM2_ESM.pdf]
