# Supplementary material for: Hepatic IRS1 and ß-catenin expression is associated with histological progression and overt diabetes emergence in NAFLD patients
Source: J Gastroenterol. 2018 May 10;53(12):1261–75. doi: 10.1007/s00535-018-1472-0 (PMC6244858; doi:10.1007/s00535-018-1472-0)
Supplement: Supplementary file 3 — Supplementary material 3 (DOCX 18 kb) [file 535_2018_1472_MOESM3_ESM.docx]

Supplementary Table 1. Relationship between blood glucose at 0, 30, 60, 120, and 180 min after an oral glucose tolerance test (OGTT) and IRS-1 and IRS-2/GAPDH ratio at 5 h post-OGTT analyzed by Spearman’s rank correlation coefficients in patients of the glucose-loaded group (N = 63)

|  | IRS-1/GAPDH ratio  at 5 h after an OGTT | | | | | IRS-2/GAPDH ratio  at 5 h after an OGTT | | | |
| --- | --- | --- | --- | --- | --- | --- | --- | --- | --- |
|  | Spearman’s rho | | *P* | | | Spearman’s rho | | *P* |  |
| Blood glucose at 0 min (mg/dL) | | −0.4152 | | 0.0001 | −0.1027 | | 0.36 | | |
| Blood glucose at 30 min (mg/dL) | | −0.4478 | | < 0.0001 | −0.0243 | | 0.83 | | |
| Blood glucose at 60 min (mg/dL) | | −0.4772 | | < 0.0001 | −0.1645 | | 0.14 | | |
| Blood glucose at 120 min (mg/dL) | | −0.2701 | | 0.015 | −0.2229 | | 0.046 | | |
| Blood glucose at 180 min (mg/dL) | | −0.2469 | | 0.017 | −0.2901 | | 0.009 | | |

Supplementary Table 2. Risk factors for developing type 2 diabetes in patients with hemoglobin A1c (HbA1c) < 6.0% at liver biopsy evaluated by univariate / multivariate Cox proportional hazard regression (N = 107)

| Parameter | Univariate | | Multivariate | |
| --- | --- | --- | --- | --- |
|  | Hazard ratio  (95% Confidence interval) | *P* | Hazard ratio  (95% Confidence interval) | *P* |
| Male | 2.01 (0.96–4.21) | 0.065 |  |  |
| Age (years) | 0.99 (0.96–1.01) | 0.28 |  |  |
| BMI (kg/m^2^) | 1.08 (1.01–1.15) | 0.019 | 1.05 (0.98–1.13) | 0.15 |
| Liver stiffness (kPa) | 0.97 (0.91–1.04) | 0.37 |  |  |
| Platelet count (×10^4^/μL) | 1.04 (0.99–1.09) | 0.14 |  |  |
| CRP (mg/dL) | 1.58 (0.68–3.70) | 0.29 |  |  |
| Albumin (g/dL) | 0.88 (0.36–2.17) | 0.79 |  |  |
| AST (U/L) | 1.01 (1.00–1.02) | 0.035 | 0.99 (0.98–1.01) | 0.34 |
| ALT (U/L) | 1.01 (1.00–1.03) | 0.0065 | 1.01 (0.99–1.02) | 0.20 |
| AST to ALT ratio | 0.39 (0.12–1.27) | 0.12 |  |  |
| GGT (U/L) | 1.00 (0.99–1.01) | 0.88 |  |  |
| Total bilirubin (mg/dL) | 0.41 (0.15–1.11) | 0.079 |  |  |
| PT-INR (%) | 0.097 (0.001–18.3) | 0.38 |  |  |
| Hyaluronic acid (ng/mL) | 1.00 (0.99–1.03) | 0.23 |  |  |
|  |  |  |  |  |
| Lobular inflammation | 1.74 (0.89–3.40) | 0.11 |  |  |
| Ballooning | 2.71 (1.43–5.13) | 0.0023 | 2.43 (1.10–5.34) | 0.027 |
| Steatosis grade | 1.64 (1.04–2.60) | 0.034 | 1.09 (0.61–1.96) | 0.76 |
| Fibrosis stage | 1.06 (0.79–1.42) | 0.72 |  |  |

Patients with HbA1c < 6.0% at liver biopsy were included in this analysis. We used univariate / multivariate Cox proportional hazard regression to estimate hazard ratios and 95% confidence intervals for diabetes risk.

AST, aspartate aminotransferase; ALT, alanine aminotransferase; BMI, body mass index; CRP, C-reactive protein; GGT, gamma-glutamyltransferase; HbA1c, hemoglobin A1c; HDL, high density lipoprotein; LDL, low density lipoprotein; OGTT, oral glucose tolerance test; PT-INR, prothrombin time-international normalized ratio.

Supplementary Table 3. Antibodies used in this study

| Antibody | Application | Description | Catalog number |
| --- | --- | --- | --- |
| β-catenin | IHC | Monoclonal mouse IgG1 | BD Biosciences, 610154 |
| IRS1 | IHC | Monoclonal mouse IgG1 | R&D Systems, MAB39781 |
| GCK | IHC | Polyclonal rabbit IgG1 | Abcam, ab70857 |

IHC, immunohistochemistry.
